# Interplay between long non-coding RNAs and epigenetic machinery: emerging targets in cancer?

**DOI:** 10.1098/rstb.2017.0074

**Published:** 2018-04-23

**Authors:** David J. Hanly, Manel Esteller, María Berdasco

**Affiliations:** 1 Cancer Epigenetics Group, Cancer Epigenetics and Biology Program (PEBC), Bellvitge Biomedical Research Institute (IDIBELL), 08908 Barcelona, Spain; 2 Department of Physiological Sciences II, School of Medicine, University of Barcelona, Barcelona, Spain; 3 Institució Catalana de Recerca i Estudis Avançats (ICREA), 08908 Barcelona, Spain

**Keywords:** non-coding RNAs, epigenetics, chromatin regulation, gene regulation, cancer

## Abstract

Of the diverse array of putative molecular and biological functions assigned to long non-coding RNAs (lncRNAs), one attractive perspective in epigenetic research has been the hypothesis that lncRNAs directly interact with the proteins involved in the modulation of chromatin conformation. Indeed, epigenetic modifiers are among the most frequent protein partners of lncRNAs that have been identified to date, of which histone methyltransferases and protein members of the Polycomb Repressive Complex PRC2 have received considerable attention. This review is focused on how lncRNAs interface with epigenetic factors to shape the outcomes of crucial biological processes such as regulation of gene transcription, modulation of nuclear architecture, X inactivation in females and pre-mRNA splicing. Because of our increasing knowledge of their role in development and cellular differentiation, more research is beginning to be done into the deregulation of lncRNAs in human disorders. Focusing on cancer, we describe some key examples of disease-focused lncRNA studies. This knowledge has significantly contributed to our ever-improving understanding of how lncRNAs interact with epigenetic factors of human disease, and has also provided a plethora of much-needed novel prognostic biomarker candidates or potential therapeutic targets. Finally, current limitations and perspectives on lncRNA research are discussed here.

This article is part of a discussion meeting issue ‘Frontiers in epigenetic chemical biology’.

## 1. Introduction

While less than 2% of the human genome has protein-coding potential, over 70% of it can undergo transcription, meaning that most of the human transcriptome consists of non-coding RNA (ncRNA) [1]. The non-coding transcriptome can be conceptualized in terms of the varying sizes of non-coding transcripts, ranging from the shorter miRNAs and piRNAs of below 40 bp, through mid-sized snoRNAs (60–300 bp) and enhancer RNAs (eRNA; 50–2000 bp) [2], to long non-coding RNAs (lncRNAs), a highly diverse group of transcripts that are all over 200 bp in length [3]. These can be further stratified into long intergenic ncRNA (lincRNAs), transcribed ultra-conserved regions (T-UCRs), natural antisense transcripts (NATs) and large intronic ncRNAs, among others. In quantity, lncRNAs outnumber protein-coding genes, yet it remains the case that relatively few of the nearly 60 000 human lncRNA genes [4] have been studied and characterized in significant detail. Nonetheless, over the past two decades, they have been linked to a wide range of molecular biological processes, ranging from nucleus-specific roles in modulating transcription, epigenetic modification, DNA looping and nuclear compartment formation, to involvement in alternative splicing and miRNA network cross-talk [5].

## 2. Long non-coding RNA cross-talk with epigenetic factors

Of the functions assigned to lncRNAs, perhaps the crucial one from the perspective of epigenetics researchers has been the hypothesis that lncRNAs directly interact with the writers, readers and erasers of histones [6]. Thus, lncRNAs may direct and determine the specific subnuclear domains and genetic loci at which the histone-modifying machinery acts in any given cell type [7]. They could also serve as molecular scaffolds of specific chromatin-regulatory complexes, combining and integrating the molecular functions of multiple histone modifiers along with other types of chromatin-regulatory machinery, thereby fine-tuning both the lower- and higher-order chromatin structure of a given target locus [8]. This section, summarized in table 1, will provide an overview of how lncRNAs shape the outcomes of gene transcription, nuclear architecture and pre-mRNA splicing.

**Table 1.** Examples of long non-coding RNAs that directly interact with histone modifiers and chromatin-associated proteins.

lncRNA	functional effects	references
Xist	histone modification complex recruitment histone modification (H3K27 deacetylation; H3K27me3; H2AK119ub) gene silencing heterochromatin formation nuclear architecture remodelling X chromosome compaction	[7–14]
HOTAIR	histone modification complex scaffolding histone modification (H3K4 demethylation, H3K27me3) gene silencing	[15–17]
ANRIL	PcG histone modification complex recruitment histone modification (H3K27me3; H2AK119ub) gene silencing	[18,19]
KCNQ1OT1	histone modification complex recruitment (G9a, PRC2) histone modification (H3K9me3, H3K27me3) locus silencing	[20,21]
AIR	G9a recruitment H3K9me3 gene silencing	[22,23]
HOTTIP	histone modification complex recruitment (WRD/MLL) histone modification (H3K4me3) gene activation	[24]
CCND1-NAT	recruitment and activation of HAT inhibitor (TLS) gene activation	[25]
ecCEBPA	DNMT1 inhibition gene activation	[26]
Dali	*trans* inhibition of DNMT1 gene activation	[27]
lincRNA-p21	hnRNP-K interaction; DNMT1 interaction p53 target gene silencing	[28,29]
Firre	nuclear architecture remodelling escape from X inactivation	[7]
MALAT1	nuclear body formation splicing factor sequestration localization to sites of active transcription	[30,31]
MIAT	Gomafu body formation splicing factor sequestration	[32–34]

## 3. Long non-coding RNA association with epigenetic modification and transcriptional regulation

The origins of the hypothesis that lncRNAs guide histone modifiers to modulate the epigenetic control of gene transcription can be traced back to earlier breakthrough studies on the process of X chromosome inactivation (XCI) [9,35]. Wutz *et al.* [10] first found that an ORFless transcript, termed Xist, is transcribed from the X-inactivation centre (Xic) and retained in the nucleus to act as the master *cis*-acting regulator of XCI. Convergently, Polycomb Repressive Complex 2 (PRC2) was found to be responsible for H3K27me3 enrichment in the Xi [36], and PRC2 recruitment to the Xi was shown to depend on Xist expression [37]. From these findings emerged the idea that Xist may guide PRC2-mediated gene repression on the Xi. Recent studies characterizing the Xist complex led to the definition of a stepwise model of the Xist molecular mechanism of XCI coordination [8]. This silencing process involves interactions with several chromatin remodelers, including the DNA-/RNA-binding protein SAFA, the binding protein SHARP and histone deacetylase 3 (HDAC3) and PRC1/PRC2 complexes [11,15,38].

As the decade progressed, interest in the idea of RNA-guided gene regulation broadened following a study on *HOX* gene transcriptional regulation [16]. Rinn *et al.* [16] demonstrated that an lncRNA transcribed from the *HOXC* locus, named HOTAIR, represses the *HOXD* locus *in trans* and in doing so recruits PRC2 to the locus to establish repressive chromatin mark H3K27me3*.* Tsai *et al.* [39] later observed that HOTAIR directly interacts not only with PRC2 at its 5′-end but also with H3K4-targeting demethylase LSD1. In parallel, two independent studies found that PRC2 alone binds to approximately 24% of transcripts genome-wide, demonstrating that a genome-wide RNA family may direct PRC2 to its target genes. These findings widened the scope of the hypothesis of lncRNA involvement in modulating chromatin modifications, establishing its potential as a global epigenetic regulatory mechanism [18,40].

Another functionally related lncRNA implicated in the epigenetic control of gene expression is known as ANRIL [19]. It is transcribed antisense to the *INK4b(p15)-ARF(p14)-INK4a(p16)* cluster of tumour suppressor genes, a locus subject to stringent regulation by both PRC1 and PRC2 components. Upon its transcription, ANRIL is thought to act in *cis* to recruit PRC2 via direct binding to the SUZ12 subunit to direct H3K27 trimethylation of the locus. ANRIL also recruits PRC1 by interacting with its CBX7 subunit, and CBX7 in turn binds H3K27me3, which stimulates H2AK199 ubiquitination by PRC1 [19,41].

The imprinted lncRNA KCNQ1OT1 was found to interact with PRC2 components EZH2 and SUZ12, as well as with G9a, to mediate in *cis* silencing of a 200 kb locus surrounding its transcription site in a lineage-specific manner [20]. Partial deletion of an 890 bp region of KCNQ1OT1 on the paternal allele, from which the lncRNA itself is exclusively expressed, resulted in the loss of paternal allele-specific gene repression of ubiquitously imprinted locus genes and reduced CpG methylation of the differentially methylated regions of these genes. It has been proposed to mediate paternal-specific Kcnq1 locus methylation by interacting with DNA methyltransferase 1 (DNMT1), as DNMT1 occupies the same ubiquitously imprinted genes and the 890 bp region of KCNQ1OT1 was shown to be necessary for co-immunoprecipitation of KCNQ1OT1 with DNMT1 [22].

lncRNA Air is another imprinted lncRNA specifically expressed from the paternal allele [25]. It is transcribed antisense to *Igf2r* and in embryos is necessary for the repression of *Igf2r* in *cis*, as well as for *Slc22a2* and *Slc22a3* silencing in the placenta. Air was found to preferentially accumulate at the *Slc22a3* promoter in mouse placental tissue [25]. It interacts with G9a, a necessary factor for *Slc22a3* silencing; thus, Air appears to recruit G9a to target H3K9 methylation at the promoter, thereby repressing *Slc22a3* expression [25].

A further locus-specific example of ncRNA-modulated gene silencing was revealed in a study by Wang *et al.* [24], in which it was found that ncRNA transcribed upstream of the *CCND1* gene upon DNA damage induction recruits TLS (Translocated in Liposarcoma) to the promoter region and allosterically releases it from its inactive conformation, enabling it to inhibit histone acetyltransferase (HAT) CBP/p300 and thus repress *CCND1* transcription [24].

As for lncRNAs with potential roles in mediating gene activation, the best example, to date, is perhaps HOTTIP, which is transcribed from the *HOXA* locus and is responsible for the activation of several *HOXA* genes through the recruitment of the WRD5/MLL histone methyltransferase (HMT) complex, which in turn facilitates gene activation by trimethylating H3K4 at the *HOXA* promoter [42]. Recently, eRNAs have also been found to interact with the CBP/p300 HAT complexes bound to enhancers, stimulating CBP HAT activity to increase H3K27ac levels at both the enhancer and the promoter of its target gene, which leads to target gene expression activation [26].

In addition to mediating histone modification, lncRNAs have also been found to directly influence DNA methylation. A novel lncRNA that is sense-transcribed from upstream of the *CEPBA* gene and that also encompasses the entire protein-coding mRNA sequence but itself remains in the nucleus has been described [28]; thereafter, it was called ‘extra-coding’ CEPBA (ecCEPBA), and it was found that it selectively inhibits DNA methylation of the *CEPBA* promoter [28]. This was due to its direct interaction with DNMT1, which has a higher affinity for ecCEBPA than for the *CEBPA* CpG island. Another group identified lincRNA-p21 as a barrier to the reprogramming of pre-induced pluripotent cells (pre-iPSCs) to iPSCs. LincRNA-p21 is induced by p53 and functions by interacting with H3K9 histone methyltransferase SETDB1 and with DNMT1. It directs these epigenetic modifiers to repress pluripotency genes by trimethylating H3K9 and methylating CpG islands within the target gene promoters [43].

A particularly unique lncRNA, telomere repeat-containing RNA (TERRA), is transcribed from telomeres and regulates telomere maintenance and function through a plurality of integrated mechanisms in a cell cycle-dependent manner (reviewed in greater depth elsewhere [44,45]). Among these mechanisms, TERRA has been proposed to act in part as a scaffold of heterochromatin-associated proteins at chromosome ends, including Shelterin complex components TRF1 and TRF2, heterochromatin protein HP1*α* and H3K9 HMT SUV39H1, in order to facilitate telomeric heterochromatin formation [30,32,46].

## 4. Long non-coding RNAs involved in nuclear architecture and three-dimensional genome structure

Recent proteomic studies of Xist interactors have also made progress in the mechanistic integration of its base-level epigenetic cross-talk with their role in the unique rearrangement of X chromosomal higher-order chromatin structure [8]. While the active X chromosome (Xa) normally assumes a distinct three-dimensional structure consisting of several topologically associated domains, Xi is compacted into two mega-domains that are fixed to the nuclear lamina, except for several mega-loops that escape XCI silencing and establish transcriptionally active foci [33]. Beginning with the establishment of gene silencing and chromatin compaction at its own locus, Xist establishes the higher-order restructuring of the Xi by first spreading to sites immediately proximal to its locus, forming the compacted, expansive Xist-coated inactive X chromatin territory that grows by drawing initially distal transcriptionally active regions into the silent compartment [33].

Another intriguing recent example of how lncRNAs can modulate the three-dimensional arrangement of the genome is *Firre*, a nuclear-retained lncRNA that localizes to chromatin both in *cis* and in *trans* to form a distinct nuclear compartment that brings into spatial proximity not only target sequences of the X chromosome but also specific loci from chromosomes 2, 9, 15 and 17 [47].

## 5. Epitranscriptomics of long non-coding RNAs—a nascent field

In a recent study providing a first glimpse of a particularly intriguing novel mechanistic layer of Xist-mediated epigenetic cross-talk, it was found that Xist reversibly undergoes N^6^-adenosine methylation (m^6^A). This mark is reportedly added by METTL3 (methyltransferase like 3), which is recruited to Xist via RNA-binding motif protein 15 (RBMP15) and RBMP15B of the Spen family. m^6^A marks on Xist are, in turn, recognized by YTHDC1 (YTH domain containing 1). All of these components appear necessary for Xist-mediated gene silencing, as loss of any of these Xist-interacting proteins leads to the failure of Xist to silence the Xi [48]. However, the mechanism by which this pathway contributes to achieve Xi silencing is unknown, let alone how it cross-talks with the other silencing factors recruited by Xist to initiate and consolidate gene silencing. Therefore, just as it will be necessary to further examine the post-translational modification dynamics of Xist-interacting proteins, so too will it also be important to find new ways of characterizing the presence and dynamics of lncRNA post-transcriptional modifications to form a more precise understanding of how lncRNA cross-talks with epigenetic modifiers.

## 6. Long non-coding RNAs associated with alternative splicing

Pre-mRNA splicing, which often occurs co-transcriptionally and *sensu stricto* post-transcriptionally, is more often than not a scenario with multiple possible outcomes that can be mechanistically determined by splice-regulatory factors, histone modifications and chromatin structure [49–51]. Although the molecular processes underlying specific types of alternative splicing event have been characterized in greater detail during the past decade, it is by no means clear how exactly a given splicing outcome is selected and set in motion for any given transcript. However, in addition to regulating the transcriptional activity of genes, lncRNAs can also influence the processing of pre-mRNA [52], and hence could be key players in determining the specific conditions under which alternative transcript isoforms are selected. The best example, to date, of the lncRNA influence on alternative splicing is given by MALAT1 (Metastasis-Associated Lung Adenocarcinoma Transcript 1), which forms a type of nuclear sub-compartment known as the nuclear speckle, and binds various splicing regulatory factors [53] such as SRSF1 [54] and serine/arginine (SR) splice-regulatory factors [54]. MALAT1 depletion leads to increased exon inclusion in most differentially spliced genes identified and showed similar alternative splicing patterns to cells with transient SRSF1 overexpression [54]. It has since been thought that MALAT1, and other splicing factor-interacting lncRNAs, negatively regulate alternative splicing events by sequestering specific splicing factors and restricting their availability to participate in alternative splicing [54,55].

An additional nuclear-retained lncRNA to have emerged during the past decade as a key non-coding player in the regulation of alternative splicing is MIAT (Myocardial Infarction-Associated Transcript) [56] and its mouse homologue Gomafu. It has been found that Gomafu contains a repeat splicing-factor 1 (SF1)-binding motif that facilitates its direct binding to this SF [57], and that oligos mimicking the Gomafu SF1-binding site delayed the onset of splicing of a weak branch point [57]. This result is consistent with the proposed role of SF1 as being required for the removal of introns with suboptimal branch points and with the notion that Gomafu restricts SF1 from interacting with sub-optimal branch points. A subsequent study of Gomafu and another splicing factor, Celf-3, provided further evidence for the idea that Gomafu may act as a molecular sponge of splicing factors to restrict their availability in the nucleus, and thereby either delay the kinetics of the splicing events that they coordinate or diminish the frequency of their occurrence [58].

## 7. Epigenetic long non-coding RNAs and cancer

Much of the impetus behind the renewed efforts of the past decade to understand the role of the non-coding genome in epigenetic cross-talk has been driven by the ongoing need to better characterize human pathologies at the molecular level [3]. Focusing on cancer, this section, summarized in table 2, highlights some key examples of disease-focused lncRNA studies that have significantly contributed to our ever-improving understanding of how lncRNAs cross-talk with epigenetic factors in health and disease, and have also provided a plethora of much-needed novel prognostic biomarkers.

**Table 2.** Examples of long non-coding RNAs associated with cancer.

lncRNA	disease	functional effects	tumorigenic effects	references
HOTAIR	breast cancer	PRC2 recruitment, chromatin modification	metastasis	[59]
	squamous cell carcinoma (SCC) (oral, oesophageal)	EZH2 recruitment histone modification (H3K27me23)	increased cell proliferation epithelial–mesenchymal transition (EMT) activation metastasis	[60,61]
	osteosarcoma	gene silencing DNA methylation regulation	cell viability evasion of apoptosis	[62]
	gastric cancer	gene silencing	EMT activation metastasis	[63]
	colorectal cancer (CRC)	PRC2 recruitment, chromatin modification (H3K27me3)	invasion and metastasis	[64]
	hepatocellular carcinoma (HCC)	EZH2 recruitment; repressor scaffolding gene silencing	increased cell viability evasion of apoptosis metastasis	[65–67]
	lung adenocarcinoma (LAD)	gene silencing	cisplatin resistance increased proliferation evasion of apoptosis	[68]
	ovarian cancer	not available	increased proliferation invasion and metastasis platinum drug resistance	[29,69]
	renal cell carcinoma (RCC)	gene activation	increased migration	[70]
	glioblastoma	PRC2 recruitment gene silencing	increased proliferation	[71]
MALAT1	lung cancer	mediation of oncogenic Oct4 transcriptional reprogramming	increased proliferation metastasis	[72–74]
	bladder cancer	SUZ12 interaction mediation of TGF-β-driven metastasis	metastasis	[75]
	RCC	EZH2 interaction; histone modification (H3K27me3)	increased cell viability evasion of apoptosis metastasis	[76]
	osteosarcoma	EZH2 interaction mediation of TGF-β-driven metastasis PI3 K/AKT pathway activation	increased cell proliferation metastasis	[77,78]
	lymphoma	EZH2 interaction, stabilization cell cycle progression	increased cell proliferation evasion of apoptosis	[79]
ANRIL	gastric cancer	PRC2 targeting histone modification (H3K27me3) gene silencing	increased proliferation evasion of apoptosis	[80]
	cervical cancer	PI3 K/AKT pathway activation	increased proliferation migration	[81]
lincRNA-p21	Pan-cancer	PRC2 targeting histone modification gene silencing cell differentiation	increased proliferation evasion of apoptosis Warburg effect	[43,82,83]
TB53TG1	CRC	protein sequestration	increased proliferation evasion of apoptosis metastasis	[84]
DACOR1	CRC	DNMT1 interaction CpG methylation gene expression regulation	increased clonogenic potential	[85]
TCF7	HCC	SWI/SNF complex recruitment nucleosome remodelling gene activation	cancer stem cell (CSC) self-renewal	[86]
ZEB2-AS	HCC	alternative splicing regulation metastasis gene regulation	metastasis increased proliferation	[12]
MIAT	chronic lymphocytic leukaemia (CLL)	not available	increased proliferation	[87]
	neuroendocrine prostate cancer (NEPC)	PcG gene network modulation	not available	[88]

One the most well-studied lncRNAs associated with cancer is HOTAIR. (A comprehensive overview of HOTAIR involvement in multiple tumour types is provided by Bhan & Mandal [89].) A pioneering metastatic lung cancer study by Gupta *et al.* [59] found that HOTAIR overexpression increased the invasive capacity of breast cancer cell lines and increased lung metastasis in mouse xenograft models. Moreover, over 800 genes were newly occupied and repressed by PRC2 following HOTAIR overexpression, including genes known to inhibit breast cancer progression. Indeed, the capacity of HOTAIR to drive breast cancer metastasis was found to depend on the concomitant presence of intact PRC2 [59]. HOTAIR has also been found to be upregulated in human oral squamous cell carcinoma samples in association with a worse prognosis score [60]. In the same study, it was found that HOTAIR enhances metastasis by repressing E-cadherin expression via its association with EZH2 [60]. HOTAIR overexpression was also connected with gastric cancer malignancy and survival outcome in a study that linked the upregulation of *HOXC* cluster genes with high-risk gastrointestinal stromal tumours [63]. A later study revealed that HOTAIR can also epigenetically regulate the transcription of miRNAs in driving gastric cancer invasion and metastasis, demonstrating that HOTAIR directly represses miR34a by PRC2 recruitment [90]. Furthermore, HOTAIR overexpression has been associated with hepatocellular carcinoma (HCC) progression [65] and its reoccurrence after liver transplantation [66]. More recently, it has been proposed that HOTAIR may act as an intermediate factor in the interaction of Snail and EZH2, facilitating Snail recruitment of EZH2 to specific genomic targets repressed by Snail. Thus, HOTAIR appears to be the linking factor of this putative Snail–HOTAIR–EZH2 tripartite epithelial–mesenchymal transition (EMT)-promoting repressor switch in HCC [67].

Some interesting examples of recent attempts to investigate the impact of HOTAIR on anti-cancer therapeutic responses can be seen in two recent studies of platinum agent-based chemotherapy resistance in ovarian cancer [29,69]. One study found that HOTAIR overexpression was associated with poor survival outcomes in patients with carboplatin-resistant ovarian tumours, and characterized an associated DNA methylation signature enriched for PcG (Polycomb Group protein) target genes that correctly, consistently and independently predicted survival outcome for carboplatin-treated patients [29]. On the other hand, the study found that patients whose tumours were ‘HOTAIR expressors’ preferentially responded to cisplatin chemotherapy, an unexpected finding that contradicted another cell-based study demonstrating that HOTAIR overexpression is associated with cisplatin resistance in an ovarian cancer cell line, and that HOTAIR knockdown sensitized the cell line to cisplatin [69].

MALAT1 owes its name to its discovery as an overexpressed transcript in non-small cell lung cancer (NSCLC) metastases that provided an early-stage marker of NSCLC metastasis risk even before its molecular functions were characterized [72]. It was later demonstrated to be a necessary factor in a mouse xenograft model of NSCLC metastasis, which was proved by employing antisense oligonucleotides (ASOs) to deplete MALAT1 in the xenograft tumours studied [73]. This also gave an early indication of the therapeutic potential of using ASOs against oncogenic lncRNAs in the treatment of lncRNA-associated disease. Its overexpression has also been linked with the invasive capacity of metastatic colorectal cancer cells [91]. Another authoritative study by Tripathi *et al.* [92] aiming to explore the mechanisms by which MALAT1 may drive oncogenesis found that MALAT1 controls cell cycle progression by modulating cell cycle gene expression, and that MALAT1 is required for the G1/S phase and mitotic progression.

Despite it being principally associated with nuclear body formation and splicing regulation at the fundamental molecular level, accumulating evidence indicates that MALAT1 overexpression contributes towards metastatic tumours predominantly through aberrantly coordinating the epigenetic regulation of gene expression [93]. Moreover, in bladder cancer, overexpressed MALAT1 promotes TGFβ-driven bladder cancer metastasis by directly interacting with PRC2 subunit Suz12 to repress E-cadherin expression [75]. Consistent with these findings, a direct MALAT1–EZH2 interaction has been implicated in renal cell carcinoma metastasis, as well as in osteosarcoma, as outlined in a recent study that directly linked the metastasis-promoting MALAT1–EZH2 interaction to E-cadherin epigenetic repression in this tumour type and established MALAT1 as a predictive biomarker of poor survival in osteosarcoma patients [77]. Furthermore, it was recently found that the association of MALAT1 with EZH2 promoted mantle cell lymphoma development, in which MALAT1 overexpression was linked with reduced overall survival. In this study, MALAT1 was found to be responsible for repressing the transcription of cyclin-dependent kinases (CDKs) p21 and p27, which are also targets of EZH2. The interaction of MALAT1 and EZH2 was also found to be enhanced by EZH2 phosphorylation at T350, and the investigators suggested that a MALAT1-mediated positive feedback loop may exist that stabilizes the interaction by promoting T350 phosphorylation of EZH2 [79].

In 2009, Guttman *et al.* [94] found a cluster of differentially expressed lncRNAs associated with the p53-mediated DNA damage response. One such lncRNA is known as lincRNA-p21, which was found to be necessary to enact the transcriptional regulation of a substantial proportion of p53 target genes [82]. lincRNA-p21 knockdown significantly increased cell viability by reducing apoptosis, whereas its overexpression increased apoptosis following DNA damage induction through direct interaction with the heterogeneous nuclear ribonucleoprotein K (hnRNP-K). [82]. hnRNP-K and lincRNA-p21 also displayed overlapping target promoter occupancy. Therefore, at least in part, lincRNA-p21 may regulate the expression of its designated branch of the p53-mediated gene regulation programme by modulating hnRNP-K localization to lincRNA-p21 target genes [82].

A more recent study revealed a novel tumour suppressor lncRNA that may constitute another important branch of the tumour suppressive p53 network [84]. In searching for novel lncRNAs epigenetically deregulated in cancer, Diaz-Lagares *et al.* first identified TP53TG1 as being CpG-hypermethylated in colon cancer cell line HCT-116. Overexpression of TP53TG1 in HCT-116 cells had a tumour suppressive effect both *in vitro* and in HCT-116-induced mouse tumours that was dependent on the presence of functional p53. Next, RNA pulldown of TP53TG1-bound proteins, combined with mass spectrometry, established that it binds YBX1, a protein with both DNA- and RNA-binding capacity that can participate directly in the regulation of gene transcription [95], pre-mRNA splicing [96] and protein translation [97]. Transfection-mediated TP53TG1 recovery in HCT116 cells reduced the level of YBX1 binding of the PI3 K promoter and, consequently, reduced the abundance of total PI3 K protein and stabilized p53 protein levels. Interestingly, hypermethylation of TP53TG1 promoter and YBX1 nuclear localization in gastric tumour samples were significantly co-associated with worse progression-free patient survival [84].

MIAT has recently been newly implicated in cancer. Sattari *et al.* [87] found that MIAT is upregulated in aggressive forms of chronic lymphocytic leukaemia (CLL). The study also revealed that MIAT is involved in a positive regulatory feedback loop with Oct4, as suppression of one led to reduced expression of the other [87], consistent with a previous study that characterized the transcriptional regulation of MIAT by Oct4 [98]. This MIAT–Oct4 regulatory loop was also found to directly contribute to apoptosis evasion in CLL, as repression of either significantly induced cell death of malignant B cells. Moreover, in an outline of emerging evidence for the general involvement of the epigenetic/non-coding interactome in neuroendocrine prostate cancer (NEPC), Crea *et al.* [88] provided preliminary evidence showing that MIAT is upregulated in aggressive NEPC and suggested that it may interact with Polycomb pathways to promote aggressive disease.

An intriguing cancer-associated example of a splice-regulatory lncRNA comes in the form of the metastasis-associated NAT of the gene encoding Zeb2, a transcriptional repressor of E-cadherin and thus a key metastasis-coordinating transcription factor. The *Zeb2* NAT, known as ZEB2-AS1, is complementary to the sequence of a large *Zeb2* intron containing an internal ribosome entry site that is necessary for its translation, and expression of this NAT promotes the retention of this intron in the mRNA by preventing it from being spliced out of the pre-mRNA, which leads to increased Zeb2 protein translation [99]. ZEB2-AS1 has recently been found overexpressed in HCC, and its downregulation in HCC reduced HCC growth and metastasis. In combination with HCC patient data analysis linking high ZEB2-AS1 expression levels with worse progression-free survival, this study indicates its potential utility as an HCC prognosis biomarker [12].

## 8. Conclusion and perspectives

Unlike genetic defects, epigenetic defects are potentially reversible and are therefore more amenable to corrective therapeutic reversal. This realization has driven the rapid emergence and expansion of epigenetic drug discovery, and drug-targeting epigenetic enzymes are in the clinic for the treatment of human disorders, especially for clinical management of haematological malignancies and neurological disorders [13,14]. However, treatments with epi-drugs still need to overcome the challenge of avoiding off-target effects. It should be considered that multiple epi-enzymes are contained in chromatin complexes, and as a result the inhibition of an epigenetic enzyme can result in changes in other members of the same protein complex. For example, treatment with the HDAC inhibitor sodium butyrate alters the acetylation level of histones, but also affects their methylation status [17]. A new layer of complexity should be taken into account, because lncRNAs could also be regulated by epigenetic mechanisms, including CpG methylation as well as histone marks [21]. The data gathered so far concerning the involvement of lncRNA in epigenetic regulation are probably still the tip of the iceberg of this emerging field, and further expansion of these data may concomitantly reveal a plethora of as-yet untapped targets and pathways amenable to epigenetic therapy. Therefore, we should consider the reactivation or silencing of specific lncRNAs after treatment with epi-drugs and the secondary effects on their target genes.

As summarized in this review, evidence of the deregulation of lncRNAs in human disorders is rapidly increasing and lncRNAs *per se* are potential targets of promising therapies. Several advantages support this research. First, lncRNAs provide a solution for targeting underexpressed epigenetic targets. While most epi-drugs produced to date are inhibitors of epigenetic enzymes, which are effective in mitigating the aberrant overexpression of epi-enzymes, success in the development of small-molecule drugs that are able to induce protein activation is very scarce. By inhibiting the expression of any given lncRNA, it is possible to achieve the upregulation of its genetic target(s) [5]. Second, and in clear contrast to short ncRNAs, lncRNA expression shows high specificity, because they are expressed at different stages of development and in a cell type-specific manner [23,27]. In addition, lncRNAs normally target one gene or a small group of related genes [5], reducing the number of non-specific targets.

Despite the great potential of targeting lncRNAs, the possibility of manipulating their expression remains largely unexplored. To date, most efforts have been focused on the silencing of specific lncRNAs using synthetic ASOs [31]. This is a very interesting strategy to follow in rare disorders that are normally caused by deficits of specific proteins. Recently, several ASO-based assays have been reported, such as the use of ASOs to re-express the paternal Ube3a allele in Angelman syndrome [34], SCN1A upregulation in Dravet syndrome [100] or the activation of Frataxin expression in Friedreich's ataxia [101]. While ASO-based lncRNA-targeting strategies are yet to reach clinical trials, MALAT1 has been the best studied lncRNA ASO target in cancer cells *in vitro* and hence is currently the most feasible lncRNA target for the development of an anti-cancer ASO therapeutic agent [73]. Moreover, if loss of function is caused by epigenetic silencing, a catalytic domain of an epigenetic enzyme fused to the CRISPR/Cas system can be used as an epigenetic editing tool to rewrite the target epigenetic mark at any particular lncRNA locus to re-express it. On the other hand, an overexpressed lncRNA could be silenced by reconfiguring its local chromatin context to repress its expression [102].

Nevertheless, many important questions regarding lncRNA complexity remain to be solved. Given their biological versatility, it is crucial to investigate whether lncRNAs are activated or silenced in any given tissue, and how they exert their respective functions. As previously described in this review, the same lncRNA can participate in several biological processes such as transcription regulation or formation of nuclear compartments. We still need to investigate how lncRNA function could be a consequence of external and internal stimuli.

In summary, the ncRNA world reveals new layers of complexity in the complex epigenetic milieu. Although we are beginning to understand their role as chromatin regulators, new functions are steadily emerging. Studies have revealed lncRNA involvement not only at the transcriptional level but also in processes such as imprinting, alternative splicing and long-range DNA looping, among others. Just as we are advancing in our understanding of lncRNA biology, new lncRNA defects associated with human disorders are being described, opening new avenues for their uses as disease biomarkers and/or therapeutic targets. However, the extent of our current knowledge is greatly limited by methodological limitations. We still need to make progress on the development of appropriate tools to equip researchers to study lncRNA structure and unravel the spatial and developmental specificities of lncRNAs.

## Data accessibility

This article has no additional data.
